# Miscellany

**Published:** 1901-07

**Authors:** 


### Miscellany.

The Teeth in Pregnancy.—In a paper published in *Obstetrics* for January, 1901, Dr. E. S. Talbot, of Chicago, used the following language:

"The old medical axiom, 'a tooth for a baby,' indicates that decay of the teeth during pregnancy has been almost universal in time and place. Decay not only takes place in new localities, but rapid disintegration about new fillings is very common. The teeth are sensitive, and so soft that they can be cut like chalk. The decay creates an appearance of horn or cartilage, showing the tooth is deprived of its lime-salts by a process similar to osteomalacia in bone."

On being questioned as to his belief in the absorption of the bone matter of the teeth during pregnancy, he claimed that he spoke of the absorption of the alveolar process and not of the teeth, and then said, "The teeth are never absorbed under such circumstances. Decay takes place from the two causes suggested, —want of vitality and autointoxication. If the teeth are kept clean, there would be no decay during pregnancy."

[We fail to see how a want of vitality or autointoxication can be causes of dental caries.—J. A. McC.]

---

Fusible Metal for Articulating Models.—Take a plaster impression of the teeth which are to articulate with the denture to be constructed. The cusps and over-bite of the anterior teeth are all that is really required in the articulating model. Such an impression can be removed without fracture in all ordinary cases. Should fracture occur, the parts can be replaced and held with a little moulding. If the teeth are isolated, it will be necessary to connect the imprints in the impression by grooves cut in the plaster, or by adding mouldine, so as to enable the fusible metal to unite them when the model is poured.

There is no need to wait for the impression to dry out. Hold the impression a few seconds in a Bunsen flame, and it is ready to receive the metal. Pour the metal when of the consistency of a stiff batter. In this state it can be built up until the model is quite thick. Pour and dip in water and separate. Ragged edges may be trimmed with a hot spatula. Five minutes is sufficient time for the entire procedure.—H. C. Wetmore, *Dominion Dental Journal.*

[The saving of time and the hardness of the model are evident advantages in the above method.—J. A. McC.]

---

Replacing Old Rubber with New in Rubber and Celluloid Dentures.—If the plate be a broken one, fasten the two parts in exact apposition with adhesive wax, run plaster in it, and flask up as for vulcanizing. Separate the flask and remove the plate from the flask and the teeth from the plate. Replace each tooth in its matrix. Pack with rubber as though it were a new case, vulcanize, and finish.

It will sometimes happen that, in repairing sets of this kind, there is such an overhanging gum that when the attempt is made to remove the plate from the plaster model the whole front part of this is broken away, rendering the model imperfect or perhaps useless. In such a case take a fine saw and cut off the gum in such a way as to leave no overhanging gum when the piece cut away is removed. The sawed part of the plate is then replaced and secured to the other part of the plate with wax. If the gum part of the plate be so thin as to be easily broken off with the fingers, they can be more accurately replaced than when the saw is used. After waxing the parts together, pour plaster in the plate and proceed as before.

The different parts of the plate can be removed from the plaster model without any danger of mutilation of the model.—T. F. CHUPEIN, *Dental Office and Laboratory.*

---

TESTING FOR DISEASE OF THE ANTRUM WITH A TUNING-FORK.—The *Medical Record* gives the following from the *Laryngoscope:* D. A. Kuyk regards the tuning-fork as a possible test for determining the presence of pus in the antrum, especially in those cases in which the usual diagnostic methods fail to give definite results. The stem of the fork should be applied, after vibration is set up, over the first or second molar. If the antra are free and clear, the tuning-fork will be heard with equal clearness on both sides and for a like duration. If one antrum contain fluid, the fork will not be heard so distinctly, perhaps very faintly, perhaps not at all,—as occurred in one case,—but, if the opposite antrum is free, the patient replies quickly and positively in the affirmative. Given another case where the symptoms are obscure, but in which trans-illumination gives a shadow on the left side and none on the right, with subjective symptoms inclining one to suspect disease of the left side, the tuning-fork placed on the left side will be heard louder and longer than on the right; the natural deduction will be that the left antral wall is thicker than the right, favoring thereby the better sound transmission, and thus almost conclusively eliminating this cavity as the offending structure. Such proved to be the case.

---

TO REMOVE TEETH FROM RUBBER PLATES.—The journals have, from time to time, offered many suggestions for this purpose. The only suggestion which we have found in part effective has been one by Dr. Genese, of Baltimore. He advises that the plate containing the teeth be placed in a saucepan and covered with glycerin, and this set over a Bunsen burner to boil. This, from the temperature it takes to boil the glycerin, will so soften the rubber that the teeth may be removed from the plate perfectly clean. But although we have tried the plan, we have not found it to be entirely effective. There will be particles of rubber clinging to the pins and next to the shoulder of each tooth. This can be removed by taking the tooth from the boiling glycerin and holding it in a rag, doubled several times, when the adhering particles of rubber around the pins may be removed with a fine excavator, for the rubber is quite softened by the heat of the boiling glycerin, and manipulated in this way gives less trouble to remove than any other way that we have tried.

The plan which we have found to leave teeth and pins absolutely free from all adhering rubber is to place the old plate in a sand-bath. This may be done in a clean ladle placed over a Bunsen burner in the chimney. The rubber will be all burnt off, while the odor of burning rubber, which is anything but pleasant, escapes up the chimney. When the rubber is all burnt off there will be a powdery residue around the pins which is readily brushed off with a stiff bench-brush.—T. F. Chupein, *Dental Office and Laboratory.*

---

An Improvised Matrix.—At a clinic given at the Ohio State Dental Society, Dr. H. M. Semans, of Columbus, demonstrated his method of adapting a simple matrix about a badly decayed lower first molar whose lingual wall was broken entirely away. The matrix of desirable thickness is cut to conform to the gum margin and bent to fit the tooth. The ends are allowed to pass a short distance beyond the remaining mesial and distal walls. A hole is punched in each end of the matrix near the gum border. One end of a thread is passed through these holes, the matrix is placed in position, and the thread is stepped over the buccal wall and forced well down on the mesial and distal sides. After tying a surgeon's knot, the ends of the thread may then be wrapped about the matrix and tooth as much as desired to secure rigidity. Rubber dam, cotton, or napkins may then be used to suit the case or the desire of the operator. A matrix put on in this way may be retained in the mouth a number of days with no discomfort to the patient.—*Dental Register.*

[In the report of this clinic no mention is made of the material out of which the matrix was formed. The writer has used a matrix in the same manner made of sheet copper of about gauge 35, B. and S. When it is annealed, it is very pliable and can be contoured.]

---

Microbic Association.—The *Peoria Medical Journal* quotes the following from Rogers, "Introduction of Medicine:"

Here is an experimental fact which is of a character to bring home the interest and importance of microbic association.

Take a culture of *bacillus prodigiosus,* a simple saprophyte, remarkable only for the beautiful red colors it gives the medium in which it develops; inject a few drops of it beneath the skin of a rabbit; no trouble whatever results. Then take a culture of *symptomatic anthrax,* an anaërobic bacillus which produces in certain animals a gaseous gangrene. The rabbit enjoys a natural immunity against this microbe, of which it can receive injections with impunity. Here, then, are two bacteria, both harmless to the rabbit.

Take now a third rabbit. Inject into it a mixture of the two cultures. Gaseous gangrene will develop and entail a speedy death. Thus two microbes which, taken separately, are harmless, occasion a deadly disease when they are united. In this instance the microbe which favors the infection—the *bacillus prodigiosus*—acts by a soluble substance, which glycerin dissolves and alcohol precipitates, which resists a temperature of 120° F., and which, by all these characteristics, resembles peptotoxin. One drop of this injected into the veins of a rabbit of two thousand grammes' weight is sufficient to abolish its natural immunity.

---

Refitting Dentures with Oxyphosphate of Copper.—At the eighth annual clinic of the Alumni Association of the Chicago College of Dental Surgery, Dr. Ames demonstrated the possibility of refitting dentures by simply placing new process oxyphosphate of copper, mixed to a stiff creamy state, upon the defective surface and pressing accurately to place, and then allowing the cement to set in contact with the tissues.

"This refit," the doctor says, "will apply to cases in which a plate has been made soon after extraction, those in which the tissues have become flabby and yielding, as well as those entire dentures in which a leak in the periphery easily admits air beneath the plate."—R. C. BROPHY, *The Bur.*

